# Simultaneous Determination of 23 Pyrrolizidine and Tropane Alkaloids in Infusions from Dry Edible Flowers Using Optimized μSPEed^®^ Microextraction Prior to Their Analysis by UHPLC-IT-MS/MS

**DOI:** 10.3390/foods13111740

**Published:** 2024-06-01

**Authors:** Begoña Fernández-Pintor, Sonia Morante-Zarcero, Isabel Sierra

**Affiliations:** 1 Departamento de Tecnología Química y Ambiental, Escuela Superior de Ciencias Experimentales y Tecnología, Universidad Rey Juan Carlos, C/Tulipán s/n, Móstoles, 28933 Madrid, Spain; begona.fernandez@urjc.es; 2 Instituto de Investigación de Tecnologías para la Sostenibilidad, Universidad Rey Juan Carlos, C/Tulipán s/n, Móstoles, 28933 Madrid, Spain

**Keywords:** dry edible flowers, infusions, tropane alkaloids, pyrrolizidine alkaloids, microextraction, HPLC–MS/MS

## Abstract

A miniaturized solid-phase extraction of two tropane alkaloids (TAs) and twenty-one pyrrolizidine alkaloids (PAs) from infusions of dry edible flowers using optimized µSPEed^®^ technique was developed. The optimization of the µSPEed^®^ methodology involved testing different cartridges and comparing various volumes and numbers of loading cycles. The final conditions allowed for a rapid extraction, taking only 3.5 min. This was achieved using a C18-ODS cartridge, conditioning with 100 µL of methanol (two cycles), loading 100 µL of the infusion sample (seven cycles), and eluting the analytes with 100 µL of methanol (two cycles). Prior to their analysis by UHPLC-IT-MS/MS, the extracts were evaporated and reconstituted in 100 µL of water (0.2% formic acid)/methanol (0.2% ammonia) 95:5 (*v*/*v*), allowing for a preconcentration factor of seven times. The methodology was successfully validated obtaining recoveries ranging between 87 and 97%, RSD of less than 12%, and MQL between 0.09 and 0.2 µg/L. The validated methodology was applied to twenty samples of edible flower infusions to evaluate the safety of these products. Two infusion samples obtained from *Acmella oleracea* and *Viola tricolor* were contaminated with 0.16 and 0.2 µg/L of scopolamine (TA), respectively, while the infusion of *Citrus aurantium* was contaminated with intermedine and lycopsamine (PAs) below the MQL.

## 1. Introduction

In recent years, the integration of edible flowers into modern gastronomy has witnessed significant growth [1,2]. The utilization of flowers is attributed, on the one hand, to their nutritional properties, as they contain bioactive compounds, such as phenolic compounds, as well as essential minerals and vitamins [3,4]. Moreover, their appeal extends to the organoleptic properties they impact to dishes, as they are typically colorful substances with a multitude of aromas and diverse textures. This is why certain chefs have embraced the inclusion of such products in their culinary creations, aiming to provide novelty and captivate the diner’s interest. In addition to being used fresh or dry as decorative elements in culinary dishes, edible flowers are also used to prepare infusions [5,6].

Numerous flowers, including rose, calendula, mallow, and hibiscus, are recognized as edible and can be categorized into three primary groups: fruit flowers, vegetable flowers and medicinal flowers [7]. Another classification criterion is based on the flowers are wild or ornamental [8]. Edible flowers intended for human consumption in the market must meet food safety standards. They should be free from pathogenic organisms and contaminants, including chemical contaminants, like pesticides [9,10]. Currently, there is no specific legislation for this type of food, though European Regulation (EU) 2015/2283, which refers to novel foods and ingredients, may encompass edible flowers [11]. The Rapid Alert System for Food and Feed (RASFF) is the organism which reports notifications about risks related to food and feed safety [12]. In the last three years, there have been some food alerts related to these edible flowers (Table S1) and the main problems are the use of unauthorized substances, such us chlorpyrifos, chlorpyrifos ethyl or thiophanate methyl, and others like the presence of some bacteria, such as *Salmonella typhimurium*. Three of the alerts were due to the use of a variety of an unauthorized flower (*Clitoria ternatea*) in some foods.

One of the main concerns associated with flower consumption is the potential presence of alkaloids. More than 12,000 alkaloids have been discovered, and they are classified as contaminants known as natural toxins. Certain plant families naturally produce them as secondary metabolites. Specifically, tropane alkaloids (TAs) represent a group of approximately 200 toxins generated by plant families such as Solanaceae, Brassicaceae, Convolvulaceae, and Erythroxylaceae. On the other hand, pyrrolizidine alkaloids (PAs) and their N-oxides (PANOs), also classified as natural toxins, constitute a group of over 660 compounds produced by families such as Boraginaceae, Orchidaceae, Asteraceae, Apocynaceae, and Fabaceae. The consumption of these toxins can produce toxic effects, such as mydriasis, tachycardia, delirium, or hallucinations in the case of TAs. PAs have been shown to be hepatotoxic, pneumotoxic, carcinogenic and genotoxic [13,14]. Both families of alkaloids can enter the food chain due to cross-contamination with plants that naturally produce these toxins, known as PA/TA-producing plants (*Datura stramonium*, *Atropa belladonna*, *Echium vulgare*, *Senecio vulgaris*, among others). Another form of contamination proposed recently is the horizontal transfer of alkaloids through the soil, so PAs/TAs produced by donor plants are taken up by the roots of acceptor plants [15,16,17,18].

Among the foods likely to contain these two families of alkaloids are teas and herbal infusions [13,14]. For this reason, Commission Regulation (EU) 2023/915 establishes the maximum TA and PA content in these products, among others. In the case of TAs, concerning herbal infusions, there is a limit for liquid infusion, which is 0.2 ng/L, considering the combined levels of atropine and scopolamine, as these are the most representative. For PAs, the limit for herbal infusion in the dry product is 200 ng/g, considering the sum of 21 PAs [19]. In tea (*Camellia sinensis*) and herbal infusions for infants and young children (liquid), the maximum level is 1 ng/mL. Given that legislative limits are set at very low levels, achieving these concentrations in food requires highly sensitive and selective methods. Moreover, due to the complexity of the samples, the sample preparation stage is typically crucial in these analyses. It enables the purification and preconcentration of the analytes to meet the established limits. Currently, the trend in method development has focused on the miniaturization and automatization of processes for enhanced environmental sustainability [20]. Another important aspect to consider is the time saved in analysis, achievable through multi-residue analyses that allow for the simultaneous determination of numerous analytes. One of the most commonly employed purification techniques for sample preparation in the determination of different contaminants in foods (e.g., antibiotics, toxins, metals, etc.) is solid-phase extraction (SPE) [21,22]. SPE can be easily miniaturized, contributing to the desired sustainability goals [23]. The term micro-SPE (m-SPE) focuses on the reduced use of sorbents, organic solvents, and sample consumption in the purification process, making the technique more environmentally friendly. For these reasons, some authors have begun to use miniaturized and automatized SPE techniques. To achieve this, various devices have been developed, such as pipette tip solid-phase extraction (PT–SPE) [24], solid-phase microextraction (SPME) [25], disposable pipette extraction (DPX) [26], stir bar sorptive extraction (SBSE) [27], microextraction by packed sorbents (MEPS) [28] or µSPEed^®^ [29]. The MEPS technique consists of a digital syringe coupled with a special cartridge called BIN (Barrel Insert and Needle) that contains a few milligrams of sorbent (1–4 mg). This syringe can be manual, semi-automatic or automatic [30]. On the other hand, µSPEed^®^ is a variant of MEPS, with the difference that it includes a unidirectional valve that allows the flow to proceed in one direction. This valve enables µSPEed^®^ to support higher pressures and ensures direct flow through the sorbent, preventing disturbances caused by solvent aspiration, as occurs in MEPS. In this context, µSPEed^®^ achieves rapid and reproducible extraction with cleaner extracts that can be directly analyzed by chromatography [31]. In this sense, some previous studies by our group have demonstrated the use of µSPEed^®^ to determine alkaloids in certain infusions [32,33]. However, to the best of our knowledge, its use for the simultaneous extraction of TAs and PAs from foods has not been optimized to date.

Until now, edible flowers represent a very novel and relatively underexplored sample. There are limited studies assessing the risk of contaminants in this food matrix. Specifically, regarding TAs, there is only one study that investigated the content of alkaloids, expressed as atropine, in pumpkin flowers using Hagner’s test, Mayer’s test, Gragendorff’s test and Wagner’s test [34]. Additionally, there are other studies that analyze PAs in this type of matrix. The first one evaluated senkirkine in *Tussilago farfara* flowers using 1% methanolic tartaric acid solution for solid-liquid extraction, followed by SPE using cation-exchange cartridges (500 mg) [35]. In the case of infusions obtained from dried edible flowers, currently there is only one study in which a microextraction procedure is performed using a C18 cartridge for the analysis of PAs in mallow, calendula, and hibiscus flower infusions [33]. To date, there are no studies of the simultaneous analysis of both families of alkaloids in edible flowers.

Therefore, the objective of this study is the development of a fast and sustainable miniaturized method using the µSPEed^®^ technique for the simultaneous analysis of TAs and PAs in infusions obtained from dried edible flowers, which have not been analyzed to date. This study will contribute to progress in controlling the presence of these natural toxins in this group of novel foods.

## 2. Materials and Methods

### 2.1. Solvents, Materials, and Standard Solutions

Methanol (MeOH) LC–MS grade, acetonitrile (ACN) LC–MS grade and dimethyl sulfoxide (DMSO) were acquired from Scharlab (Barcelona, Spain). Ammonia solution (25%) LC–MS grade was purchased from Merck KGaA (Darmstadt, Germany) and formic acid (FA) LC–MS grade was purchased from Fischer Scientific (Loughborough, UK). A Millipore Milli-Q system (Billerica, MA, USA) was used to obtained ultrapure water (H_2_O) (18.2 MΩ cm).

The standards of atropine (≥99%), scopolamine hydrobromide (≥98%) and retrorsine (≥90%) were acquired from Sigma-Aldrich (St. Louis, MO, USA). The rest of the PAs and PANOs, echimidine perchlorate (≥90%), echimidine *N*-oxide (≥95%), europine hydrochloride (≥98%), europine *N*-oxide (≥98%), heliotrine (≥90%), heliotrine *N*-oxide (≥95%), intermedine (≥98%), intermedine *N*-oxide (≥95%), lasiocarpine (≥95%), lasiocarpine *N*-oxide (≥95%), lycopsamine (≥95%), lycopsamine *N*-oxide (≥90%), retrorsine *N*-oxide (≥95%), seneciocine (≥98%), senecionine *N*-oxide (≥95%), seneciphylline (≥98%), seneciphylline *N*-oxide (≥98%), senecivernine (≥95%), senecivernine *N*-oxide (≥98%) and senkirkine (≥95%), were purchased from PhytoLab GmbH and Co. KG (Vestenbergsgreuth, Germany). Individual standard solutions (1000 mg/L) of the analytes were prepared in amber vials according to their solubility. TAs (atropine and scopolamine) and PAs (europine, heliotrine, europine *N*-oxide, heliotrine *N-*oxide, intermedine, senecionine, lycopsamine, retrorsine, and seneciphylline) were prepared in ACN/DMSO (4:1, *v*/*v*) and the rest (echimidine *N*-oxide, lasiocarpine *N-*oxide, echimidine, intermedine *N*-oxide, lycopsamine *N-*oxide, senecivernine, lasiocarpine, retrorsine *N*-oxide, seneciphylline *N*-oxide, senecionine *N*-oxide, senkirkine and senecivernine *N*-oxide) were prepared in MeOH. A solution containing the two TAs (1 mg/L) was prepared in MeOH with the appropriate dilution of the individual standards and a solution containing the 21 PAs (1 mg/L) was prepared in MeOH. The standard solutions were stored in darkness at −18 °C.

For the µSPEed^®^ extraction, a digiVol digital syringe of 500 µL and octadecylsilane (C18-ODS, 4 mg, 3 µm, 120 Å) and porous cross-linked polystyrene divinylbenzene (PS/DVB, 4 mg, 3 µm, 120 Å) cartridges were acquired from EPREP (Mulgrave, VIC, Australia).

### 2.2. Samples and Infusion Preparation

**Figure 1:**
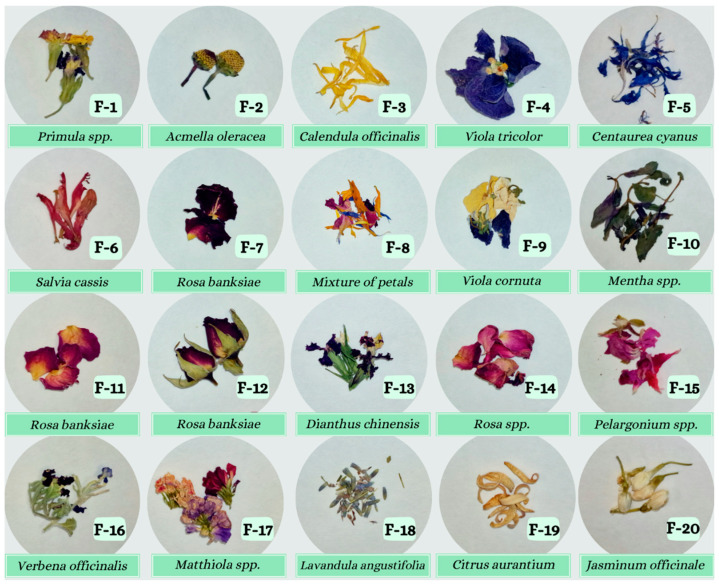
Dry edible flowers (scientific names) used for infusion preparation.

Dried edible flowers (see Figure 1), including primrose (*Primula* spp., Primulaceae), toothache plant (*Acmella oleracea*, Asteraceae), pot marigold (*Calendula officinalis*, Asteraceae), Johny jump up (*Viola tricolor*, Violaceae), cornflower (*Centaurea cyanus*, Asteraceae), salvia (*Salvia cassis*, Lamiaceae), lady banks with red and pink petals (*Rosa banksiae*, Rosaceae), horned violet (*Viola cornuta*, Violaceae), mint and chocolate (*Mentha* spp., Lamiaceae), lady banks full flower (*Rosa banksiae*, Rosaceae), rainbow pink (*Dianthus chinensis*, Caryophyllaceae), rose petals (*Rosa* spp., Rosaceae), geranium (*Pelargonium* spp., Geraniaceae), common vervain (*Verbena officinalis*, Verbenaceae), stock (*Matthiola* spp., Brassicaceae), English lavender (*Lavandula angustifolia*, Lamiaceae), bitter orange (*Citrus aurantium*, Rutaceae), jasmine (*Jasminum officinale*, Oleaceae) and a mixture of petals, were purchased at different stores in Spain. In that sense, infusions from the dried edible flowers were obtained following the International Standard ISO 3103 protocol [36]. For this purpose, 2 g of each edible flower were weighed using an analytical balance (±0.01 g) and infused in 100 mL of boiling Milli-Q H_2_O (100 °C), allowing them to brew for 5 min with a tap. Subsequently, the infusion samples were filtered through a 0.45 µm PTFE filter membrane. Sampling was conducted in accordance with the European Commission Regulation No. 401/2006 [37]. Accordingly, 3 sub-samples were collected, with each sub-sample infused in triplicate and each infusion extract subjected to triplicate analysis (*n* = 9).

### 2.3. µSPEed^®^ Extraction

The optimization of the extraction procedure for PAs and TAs from the infusions of edible flowers was conducted by testing cartridges with different packing materials (PS-DVB and C18-ODS) and extraction conditions (number and volume cycles). This was performed using a blank sample (*Jasminum officinale*) spiked with a standard solution containing all the analytes at a concentration of 0.2 ng/mL. Under the optimized conditions, the microextraction of the target analytes from the flower infusions was conducted using the µ-SPEed^®^ syringe with the C18-ODS sorbent using the extract-discard mode. The procedure involved conditioning the sorbent with two cycles of 100 µL of MeOH followed by two cycles of 100 µL of acidified water (0.1% FA). Subsequently, for sample loading, seven cycles of 100 µL of the infusion extract were passed through the cartridge with no washing step. Finally, the analytes were eluted from the sorbent with 200 µL of MeOH into an Eppendorf^®^. The extract was evaporated and reconstituted with 100 µL of mobile phase (H_2_O (0.2% FA)/MeOH (0.2% ammonia) 95:5 (*v*/*v*)) and, finally, it was analyzed by UHPLC-IT-MS/MS. The flow rate was set at 15 μL/s for the MeOH steps and 5 μL/s for the H_2_O steps throughout all the assays.

### 2.4. UHPLC-IT-MS/MS Analysis

The analysis of the purified extracts of the edible flower infusions were carried out with an UHPLC system (Dionex UltiMate 3000, Thermo Scientific, Waltham, MA, USA). The chromatographic system was coupled to an ion-trap tandem mass spectrometer detector (ESI-ITMS amaZon SL, Bruker, Billerica, MA, USA). The separation was performed using a Luna Omega Polar C18 column (1.6 μm particle size, 100 mm × 2.1 mm) purchased from Phenomenex, Torrance, CA, USA) at 30 °C. The separation was carried out following a methodology previously developed by the research group using a mobile phase gradient elution with MeOH with 0.2% ammonia (solvent A) and H_2_O with 0.2% FA (solvent B) as follows: the gradient starts with 5% of solvent A and this proportion is maintained during 0.5 min, then increases from 5 to 10% of A in 3 min, from 10 to 25% of solvent A in 4 min, from 25 to 30% of A in 2 min, from 30 to 70% of solvent A in 3 min and, finally, from 70 to 5% in 2 min, constituting a total run time of 14.5 min with a flow rate of 0.3 mL/min. Then, a final minute of re-equilibration occurs to return to the initial conditions. The injection volume used was 5 μL [38].

The parameters for the mass spectrometry detection were set as follows: the electrospray ionization interface (ESI) operated in positive ion mode, the ion spray voltage was 4500 V, the end plate offset was 500 V, the nebulizer gas was 20 psi and the dry gas flow rate was 10 L/min with a temperature of 200 °C. Multiple reaction monitoring (MRM) scan mode was used for the analytes, and the mass spectrum parameters of each analyte were obtained by direct infusion of individual standard solutions of each TA and PA (5 μg/mL) at a flow rate of 4 μL/min. The precursor ion of each analyte ([M + H]^+^) was identified and subsequently isolated and fragmented to acquire the mass spectrum (MS^2^) with the product ions of each analyte (see Table S2). Regarding each analyte, the most intense product ion of the MS^2^ spectrum was chosen for quantification, while the remaining ions (with at least one being obligatory) were employed for confirmation.

### 2.5. Method Validation

The proposed µ-SPEed^®^-UHPLC-IT-MS/MS methodology was validated in terms of accuracy, precision, linearity, selectivity, matrix effects (ME), method detection (MDL) and quantification (MQL) limits. These parameters were assessed according to the criteria established in the European Commission SANTE/11312/2021 document for pesticides [39]. The validation was performed at four levels of concentration with the *Jasminum officinale* infusion sample. According to the legislation, the maximum concentration of TAs allowed for liquid infusions is 0.2 ng/mL, so this concentration was set as one level of validation. Based on this value and the limits of quantification of the analytes, 0.1 ng/mL was set as the second level of validation. Finally, 2 ng/mL and 10 ng/mL were selected as the third and fourth levels of validation to cover concentration values that are above the legislated limit. In the case of PAs, regulations have set a limit of 200 ng/g for dried products destined for herbal infusions, such as edible dried flowers. Hence, considering the amount of dried flower used for the infusions (2 g) and the H_2_O volume of infusions (100 mL), 200 ng/mL corresponds to a concentration of 4 ng/mL in the liquid, and this concentration was established as intermediate level for the methodology validation. Additionally, based on this value and the limits of quantification of the analytes, a compromise was reached to choose 0.2 ng/mL (corresponding to 10 ng/g) as the low validation level. Finally, 4 ng/mL (corresponding to 200 ng/g) and 20 ng/mL (corresponding to 400 ng/g) were taken as the third and fourth validation levels, respectively.

The linearity of the method was evaluated with the matrix-matched calibration curves. For this purpose, the curves were prepared at six known concentration levels from 0.1 ng/mL to 50 ng/mL for TAs and from 0.2 ng/mL to 50 ng/mL for PAs. These concentration levels were obtained by spiking extracts of the *Jasminum officinale* infusion sample, after the purification procedure, with standard solutions at different concentrations of the target compounds. According to the validation guidelines, the coefficient of determination (R^2^) value should be close to 1 to ensure good linearity. Additionally, to determine the ME, solvent-based calibration curves, not subjected to the µSPEed^®^ procedure, were prepared using standard solutions at the same concentration levels as the matrix-matched calibration curves. The ME was calculated by comparing the slopes of the calibration equations obtained for each analyte from both matrix-matched and solvent-based calibration curves, both expressed in ng/mL (slope matrix-matched/slope solvent-based × 100). The selectivity is related to the mass spectra of the target analytes obtained from sample extracts in comparison with the mass spectra obtained in the standard solutions.

The sensitivity of the method is related to the MDLs and MQLs, which were calculated based on the signal-to-noise ratio (S/N) provided by the UHPLC–IT–MS/MS from the extracted ion chromatograms of the edible flower infusion extract at the low level of concentration. Consequently, the concentrations corresponding to a S/N of 3 and 10 were considered for the MDLs and MQLs, respectively, both expressed in ng of PAs/TAs per mL of edible flower infusion.

The accuracy was evaluated at the four concentration levels in terms of recovery. The study was performed by spiking the edible flower infusion with a standard solution containing the analytes and then subjecting them to the extraction process. The areas were compared with those obtained from the analysis of simulated extracts (non-spiked infusion samples subjected to the extraction procedure and spiked after the extraction). The results were expressed as the mean recovery obtained from 9 samples extracted in different days. The precision was evaluated at the four validation levels in terms of relative standard deviation percentage (RSD %). Repeatability (intra-day precision) was evaluated by analyzing on the same day six replicate extracts (*n* = 6) of an infusion sample, while reproducibility (inter-day precision) was evaluated analyzing three replicate extracts over three different days of an infusion sample (*n* = 9).

## 3. Results

### 3.1. µSPEed^®^ Extraction Optimization

To optimize the simultaneous extraction of 23 PAs and TAs, different studies were conducted. C18 silica-based and PS-DVB polymeric-based sorbents were evaluated, testing different load volumes and number of cycles. As a starting point, the optimized conditions established in various methodologies previously developed by our research group for the extraction of 2 TAs [32] or 21 PAs [33] were taken as a reference.

Firstly, PS–DVB cartridges were evaluated by loading 5 cycles of 500 µL of the jasmine infusion sample (0.2 ng/mL) and eluting with 2 cycles of 100 µL of MeOH. As shown in Figure S1a, the results obtained were not satisfactory. This could be attributed to the competitive effect among target analytes for the available adsorption sites, due to the higher number of alkaloids (23 instead of 2). For best results, this procedure was replicated by reducing the number of loading cycles (3 and 2 cycles) while maintaining the loading volume (500 µL). However, the extraction recoveries did not increase significantly. Then a second strategy was tried, reducing the sample amount to 100 µL, with 3 loading cycles, and eluting with 100 µL of MeOH. As can be seen in Figure S1b, the recoveries of the 23 target analytes were higher that 70%, confirming the π–π interactions between the polymeric sorbent and the aromatic rings of the alkaloids. Therefore, to achieve higher preconcentration factor, 5 and 7 cycles of 100 µL of the infusion sample were tested, but a decrease in recoveries was observed (Figure S1b). This decrease in recovery could also be attributed to a potential washout effect by increasing the number of cycles, as there are numerous analytes, and the cartridge has a limited amount of sorbent.

**Figure 2:**
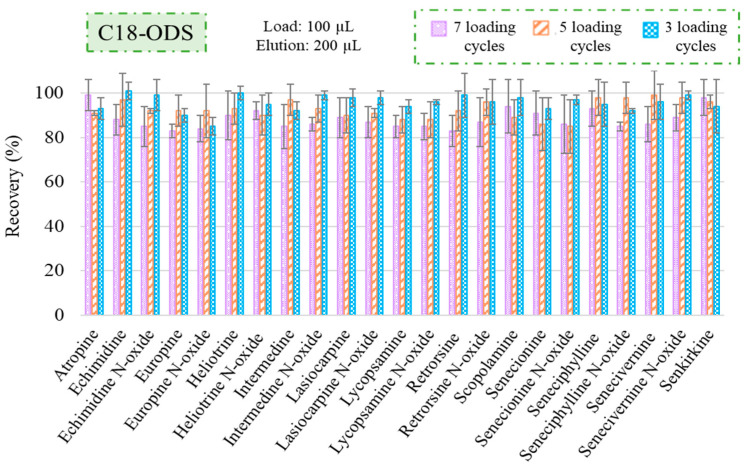
Recovery percentages (*n* = 3) for TAs and PAs with C18-ODS cartridges after carrying out the µSPEed^®^ extraction using a jasmine flower infusion spiked with 0.2 ng/mL of a standard solution containing the target analytes, loading 100 µL of sample, 7, 5 and 3 cycles.

Subsequently, the C18-ODS cartridges were evaluated according to Casado et al. [33]. The sorbent was conditioned with two cycles of 100 µL of MeOH followed by two cycles of 100 µL of H_2_O. Then, 3 cycles of 100 µL of the infusion sample were used for the loading step and analytes were eluted with 100 µL of MeOH. As shown in Figure 2, the recoveries obtained under these conditions were satisfactory for the 23 target analytes, confirming that this sorbent provides strong hydrophobic interactions with the alkaloids. Subsequently, some assays were carried out by increasing the number of cycles to achieve a higher preconcentration factor, so 5 and 7 loading cycles were tested. However, in these experiments, the conditioning step of the cartridge was modified, and 100 µL of acidified water (0.1% FA) was used after the 2 cycles of MeOH. This conditioning strategy improved the sample flow through the cartridge and avoided overpressures that can occur when working with aqueous media, thus avoiding potential workflow impediments. Additionally, the elution step was modified, and it was carried out in 2 cycles of 100 µL of MeOH to ensure that analytes were eluted completely. Finally, to obtain a higher preconcentration factor, the extracts were evaporated and reconstituted in 100 µL of mobile phase (H_2_O (0.2% FA):MeOH (0.2% ammonia)) (95:5, *v*/*v*) because it was observed that this solvent enhances the resolution of the chromatographic peaks. As is shown in Figure 2, good results were obtained under these conditions with recoveries higher than 85% for the target analytes. Finally, a study was conducted increasing to 10 loading cycles to verify if the preconcentration factor could be increased further. However, in this case, the recoveries decreased drastically, showing percentages below 50%. Therefore, this condition was discarded, prompting the selection of 7 cycles as optimal sample loading to achieve the highest preconcentration factor.

**Figure 3:**
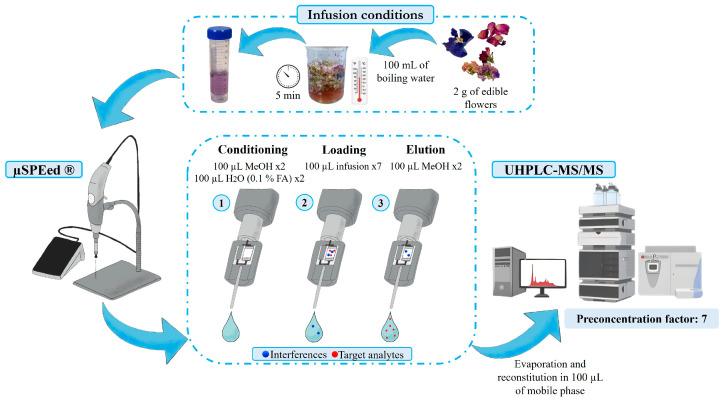
Infusion preparation from dry edible flowers and optimized µSPEed^®^ extraction procedure for TAs and PAs prior to their analysis by UHPLC–IT–MS/MS.

Based on the results obtained, the final optimized conditions for the µSPEed^®^ procedure were C18-ODS cartridges conditioned with 100 µL MeOH (2 cycles), followed by 100 µL of acidified water (0.1% FA), loading 100 µL of the infusion sample (7 cycles), eluting the analytes with 100 µL of MeOH (2 cycles) (see Figure 3). This methodology allowed the simultaneous analysis of 23 natural toxins belonging to the two families (TAs and PAs), which represents an advantage compared to the methods previously developed for the individual analysis of these families.

### 3.2. Evaluation of the “Greenness” of the Optimized Microextraction Methodology

Nowadays, sample preparation is a crucial step in Green Analytical Chemistry (GAC). The methodologies developed in line with this concept aim to minimize the use of solvents and toxic reagents, as well as reduce waste and energy consumption. A strategy in GAC involves the miniaturization and automatization of sample preparation that can be achieved with a procedure based on μSPEed^®^. In this work, the “greenness” of the optimized methodology was evaluated using the AGREEprep tool, which assesses the sample preparation stage following the ten principles of the Green Sample Preparation (GSP): (1) the sample preparation placement, (2) the use of hazardous materials and sustainability, (3) the renewability and reusability of materials, (4) the wastefulness of the methods, (5) the size of the sample, (6) the sample throughput, (7) the integration and automation, (8) the energy consumption, (9) the post-sample preparation analysis and (10) the operator’s safety [40]. Table 1 shows the scores assigned to each point and the corresponding weight applied.

**Figure 4:**
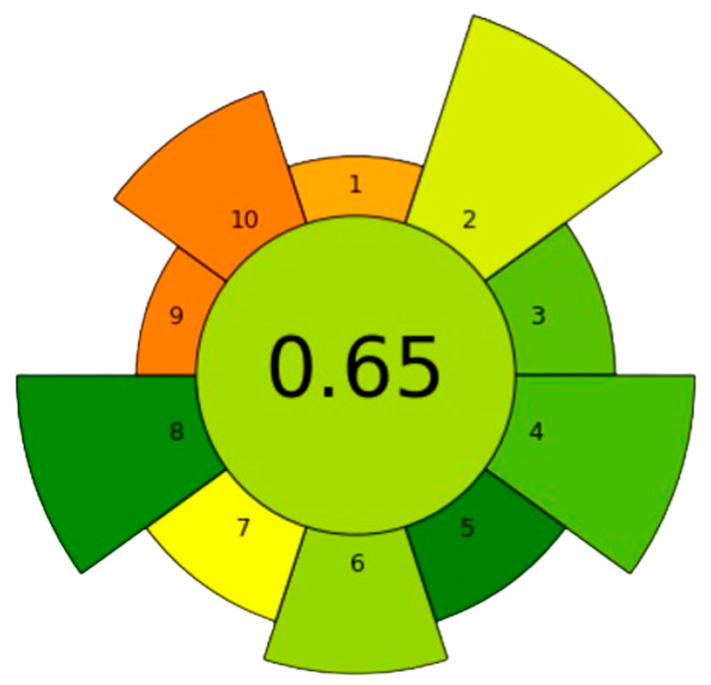
Results of AGREEprep assessment of µSPEed^®^-UHPLC–IT–MS/MS method for determination of 23 pyrrolizidine and tropane alkaloids in dry edible flower infusions.

Two of the lowest scores were attributed to sample preparation placement and post-sample preparation analysis; however, these aspects were given minimal weight because they are essential in food analysis, as legislation mandates their use to analyze contaminants in this matrix. Another input that received a significant penalty was operator’s safety because MeOH was used for conditioning of the cartridges and elution of the analytes, and this solvent bears three pictograms on its label. The remaining aspects analyzed by the system received good scores, highlighting the limited use of hazardous materials (0.3 mL), low waste of the method, minimal use of sample (0.014 g per extraction), high sample throughput (17 samples per hour), and low energy consumption (17.7 Wh). One advantage of this technique is the reusability of the material, which can be reused more than 30 times without any decrease in recovery percentages. This feature represents a sustainable approach. Figure 4 shows the pictogram obtained after evaluating the previously optimized methodology. The score of 0.65, displayed in green, suggests that the methodology developed is indeed a sustainable approach.

### 3.3. Method Validation

The validation of the method was carried out with the jasmine infusion sample according to Section 2.5 and the result are presented in Table 2.

**Figure 5:**
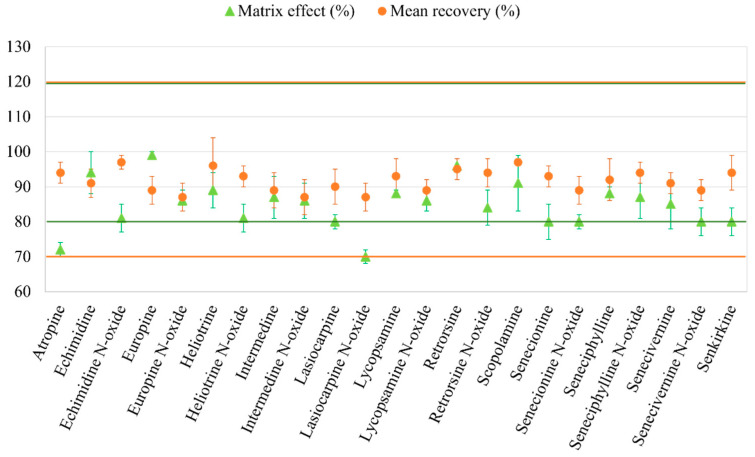
Matrix effect (%) and mean recovery (%) for the TAs and PAs with the µSPEed^®^-UHPLC–IT–MS/MS method under the optimized conditions. Matrix effect (ME) calculated as the ratio (slope matrix-matched/slope solvent-based) × 100. Green lines indicated the limits of the ME and orange lines indicates the limits of the recovery percentages as indicates the European Commission SANTE/11312/2021 document.

As can be observed, the linear regression was good for the 23 alkaloids. For the TAs, the linear range was evaluated between 0.1 and 10 ng/mL and a coefficient of determination (R^2^) up to 0.997 was obtained. For the PAs, the R^2^ ranged between 0.993 and 0.999 and the linear range studied was between 0.2 and 20 ng/mL. Regarding the MDL, for both TAs (atropine and scopolamine) it was 0.03 µg/L and for the 21 PAs it was between 0.03 and 0.07 µg/L. The MQL was 0.1 µg/L for the TAs and between 0.09 and 0.2 for the PAs. The ME was evaluated, and percentages below 100% indicate signal suppression, while percentages higher than 100% mean a signal increase. If the percentage is between 80 and 120%, ME can be ignored, and solvent-based calibration curves can be used for analyte quantification. However, if the percentage is outside this range, matrix-matched curves must be considered in calibration. Figure 5 shows that the analytes that experienced a significant suppression of the signal were the atropine and lasiocarpine *N*-oxide. All the other analytes did not show ME, which represented an advantage because these analytes do not exceed the limits established by the validation guide (80–120%) and the solvent-based curves can be used to quantify these analytes in the flower infusions. The accuracy and precision were evaluated at the four concentration levels selected, as explained in Section 2.4. The results show good accuracy at all the levels with recoveries ranging between 94 and 97% for TAs and 87 and 97% for Pas and, as can be seen in Figure 5, all the analytes were between the range established by the validation guide (70–120%). In addition, precision also showed satisfactory results since RSD ≤ 10% were obtained for TAs and ≤12% for PAs for the intra-day and inter-day precision, values below the 20% established by the validation guide. All of these parameters confirm the successfully validation of the proposed methodology [39].

**Figure 6:**
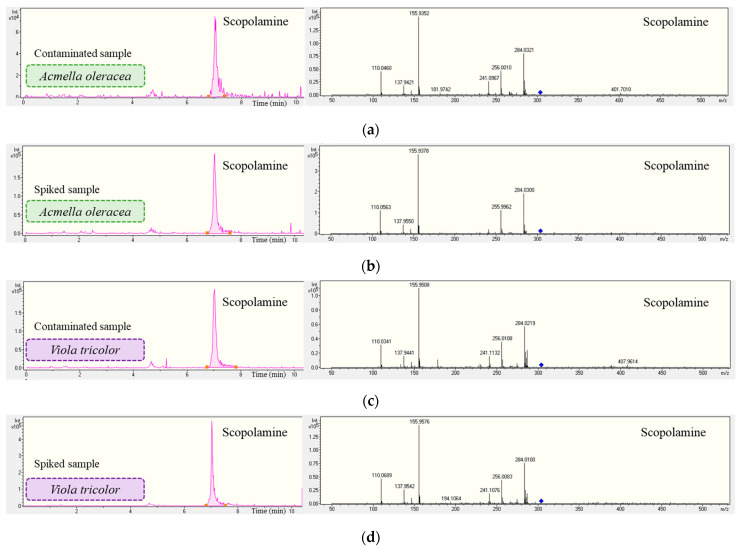
Extracted ion chromatogram and mass spectrum (MS^2^) of scopolamine in infusions of (**a**) contaminated Acmella oleracea, (**b**) contaminated Acmella oleracea spiked at 0.2 ng/mL, (**c**) contaminated *Viola tricolor* sample, (**d**) contaminated *Viola tricolor* spiked at 0.2 ng/mL.

The selectivity of the method was evaluated verifying the deviation of the retention time in comparison with the standard solution chromatogram. It was observed that no deviation exceeded 2.5 min, as specified the validation guide. Additionally, the ion transition ratios in the contaminated samples were examined, ensuring that they did not deviate by more than 30% in relative abundance compared to spiked samples [39]. Figure 6 shows the extracted ion chromatogram and the mass spectrum for the scopolamine in Acmella oleracea and *Viola tricolor*, since both samples were contaminated with this toxin. It can be seen that the retention time and relative abundance did not vary more than the criteria mentioned previously.

### 3.4. Analysis of Real Samples

The validated µSPEed^®^-UHPLC–IT–MS/MS method was applied to 20 infusions from dry edible flowers to evaluate the safety of these products. These infusions were prepared in triplicate and analyzed as previously explained in Section 2.2 and Section 2.3. The contaminated samples were quantified using their corresponding matrix-matched curves.

**Figure 7:**
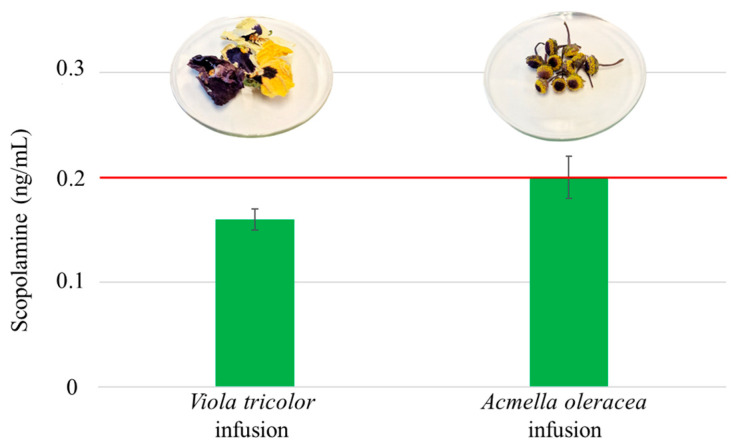
Scopolamine quantified in infusions of dry edible flowers analysed (*Acmella oleracea* and *Viola tricolor*). Red line shows the limit established by the Commission Regulation (EU) 2023/915.

Regarding the results obtained, 17 samples were below the MDL, 1 sample (*Citrus aurantium*) contained lycopsamine and intermedine, but it could not be quantified as the result was below the MQL, and 2 samples (Acmella oleracea and *Viola tricolor*) were contaminated with 0.2 ± 0.1 and 0.16 ± 0.01 ng/mL of scopolamine, respectively. In the case of Acmella oleracea, it belongs to the Asteraceae family, known for producing alkaloids as a defense mechanism. However, *Viola tricolor* belongs to the Violaceae family, which is not a productor of alkaloids. The concentrations found in this sample could be due to cross-contamination with TA-producing plants or the presence of alkaloids in the soil in which they were grown. In fact, some studies have demonstrated the horizontal transfer of the alkaloids through the soil, so this could be the reason of the presence of this toxin in the sample [18]. In the analyzed samples belonging to the Asteraceae family, such as Calendula officinalis, it was expected to obtain positive samples in PAs since some authors found these toxins in this flower [33,41]. However, the absence of these compounds in the analyzed infusion may be due to the low transfer rate during the brewing process. In that respect, Fernández-Pintor et al. [42] carried out a study to determine the transfer rate of some PAs during the brewing of herbal infusions contaminated with Echium vulgare and Senecio vulgaris weeds. In this work, it was confirmed that the transfer is very low for some (e.g., senkirkine, seneciphylline and seneciphylline *N*-oxide). Therefore, there is a possibility that these analytes were not transferred to the infusion, which could explain why they are not detected [42]. As mentioned previously, Commission Regulation (EU) 2023/915 has set a limit of 0.2 ng/mL for the TAs (as the sum of atropine and scopolamine) in infusions. Figure 7 shows that the infusion of Acmella oleracea was contaminated at a concentration within the limit set by the legislation.

## 4. Conclusions

In this work, a miniaturized µSPEed^®^ procedure was developed to purify edible flower infusions for the simultaneous analysis of 2 TAs and 21 PAs. This methodology allows for the reduction of the solvents, as it only requires 400 µL of methanol and 700 µL of sample, which represents a sustainable approach. Additionally, it contributes to the reduction of the analysis time because it analyzes two families of toxins that must be studied according to legislation. The procedure was successfully applied to twenty real samples, revealing the presence of scopolamine in two of them (*Viola tricolor* and *Acmella oleraceae*) and intermedine and lycopsamine was detected in one (*Citrus aurantium*). The results obtained, along with the limited methodologies and studies available nowadays, confirm that further studies should be carried out on this food matrix. Ensuring the safety of this group of novel foods is crucial to prevent any potential risk to human health.

## Figures and Tables

**Table 1:** Input used to assign AGREEprep scores for µSPEed^®^-UHPLC-IT-MS/MS method.

Criterion	Input	Justification for Input	Weight	Score
Sample preparation placement	On site	Although the sample preparation is performed in the laboratory, it could be possible to perform this on site because of the versatility and flexibility of the SPE procedure	1	0.33
Hazardous materials	0.3002	0.2 mL of MeOH (conditioning) per sample + 0.002 mL formic acid (conditioning) + 0.1 mL (mobile phase to reconstitute the extract after the evaporation step)	5	0.57
Sustainability, renewability, and reusability of materials	>75% of reagents and materials are sustainable or renewable	Water is the solvent most commonly used in this methodology, which implies more than 75% of sustainable and renewable materials. SPE cartridges are not sustainable, but they can be used several times. The only reagent not sustainable is MeOH	2	0.75
Waste	0.418	0.004 g of C18-ODS, 0.2 mL of MeOH (conditioning), 0.2 mL of H_2_O (1% formic acid) and 0.014 g of sample	4	0.77
Size economy of the sample	0.014	Amount of dry edible flower (g) used for one extraction	2	1.00
Sample throughput	17	3.5 min per µSPEed^®^ extraction (60/3.5 = 17 samples/h)	3	0.67
Integration and automation	2 steps, semi-automated system	µSPEed^®^ extraction and evaporation of the extract	2	0.50
Energy consumption	11.7 Wh per sample	µSPEed^®^ extraction (60 W) for 3.5 min and evaporation of the sample extract (245 W) for 2 min	4	0.96
Post-sample preparation configuration for analysis	Liquid chromatography	UHPLC–MS/MS	1	0.25
Operator’s safety	3 hazards	3 pictograms for the MeOH	3	0.25

**Table 2:** Validation parameters of the optimized µSPEed^®^-UHPLC–IT–MS/MS method proposed for the determination of PAs and TAs in Jasminum officinale (jasmine) infusion sample.

Analytes	Matrix-Matched Calibration R^2^	Accuracy		Precision		MDL * (µg/L)	MQL * (µg/L)	ME (%)
		Recovery (% ± SD)	Mean Recovery (% ± SD)	Intra-Day (RSD %)	Inter-Day (RSD %)			
Atropine	y = 316,854x − 1,985,075 0.998	94 ± 10 ^a^	94 ± 3	6 ^a^	10 ^a^	0.03	0.10	72
		90 ± 7 ^b^		6 ^b^	7 ^b^			
		98 ± 5 ^c^		10 ^c^	5 ^c^			
		95 ± 7 ^d^		6 ^d^	8 ^d^			
Echimidine	y = 511,966x + 2,223,340 0.996	97 ± 5 ^a^	91 ± 4	9 ^a^	6 ^a^	0.04	0.11	94
		90 ± 7 ^b^		9 ^b^	7 ^b^			
		87 ± 7 ^c^		7 ^c^	8 ^c^			
		89 ± 8 ^d^		4 ^d^	9 ^d^			
Echimidine *N*-oxide	y = 831,663x + 2,100,641 0.999	95 ± 7 ^a^	97 ± 2	10 ^a^	8 ^a^	0.03	0.09	81
		96 ± 8 ^b^		6 ^b^	9 ^b^			
		100 ± 5 ^c^		3 ^c^	5 ^c^			
		98 ± 4 ^d^		4 ^d^	4 ^d^			
Europine	y = 420,430x + 2,347,655 0.995	94 ± 8 ^a^	89 ± 4	9 ^a^	8 ^a^	0.07	0.20	99
		90 ± 6 ^b^		10 ^b^	7 ^b^			
		89 ± 8 ^c^		3 ^c^	9 ^c^			
		84 ± 4 ^d^		3 ^d^	4 ^d^			
Europine *N*-oxide	y = 556,541x + 3,751,797 0.993	91 ± 5 ^a^	87 ± 4	7 ^a^	6 ^a^	0.07	0.20	86
		86 ± 8 ^b^		6 ^b^	10 ^b^			
		82 ± 5 ^c^		6 ^c^	7 ^c^			
		89 ± 9 ^d^		2 ^d^	11 ^d^			
Heliotrine	y = 422,499x + 1,232,609 0.997	84 ± 5 ^a^	96 ± 8	11 ^a^	6 ^a^	0.07	0.20	89
		98 ± 7 ^b^		9 ^b^	7 ^b^			
		100 ± 8 ^c^		4 ^c^	8 ^c^			
		100 ± 4 ^d^		6 ^d^	4 ^d^			
Heliotrine *N*-oxide	y = 375,336x + 993,904 0.999	89 ± 7 ^a^	93 ± 3	5 ^a^	7 ^a^	0.06	0.17	81
		95 ± 5 ^b^		4 ^b^	6 ^b^			
		96 ± 10 ^c^		5 ^c^	10 ^c^			
		91 ± 7 ^d^		5 ^d^	8 ^d^			
Intermedine	y = 228,602x + 1,039,467 0.997	95 ± 4 ^a^	89 ± 5	9 ^a^	4 ^a^	0.07	0.20	87
		90 ± 9 ^b^		6 ^b^	10 ^b^			
		82 ± 6 ^c^		4 ^c^	7 ^c^			
		88 ± 5 ^d^		3 ^d^	6 ^d^			
Intermedine *N*-oxide	y = 255,102x + 1,200,504 0.997	84 ± 9 ^a^	87 ± 5	7 ^a^	10 ^a^	0.07	0.20	86
		89 ± 8 ^b^		8 ^b^	9 ^b^			
		81 ± 9 ^c^		7 ^c^	11 ^c^			
		92 ± 7 ^d^		6 ^d^	7 ^d^			
Lasiocarpine	y = 1,189,304x − 3,348,955 0.995	84 ± 10 ^a^	90 ± 5	10 ^a^	12 ^a^	0.06	0.17	80
		94 ± 5 ^b^		3 ^b^	6 ^b^			
		94 ± 8 ^c^		6 ^c^	8 ^c^			
		88 ± 4 ^d^		5 ^d^	5 ^d^			
Lasiocarpine *N*-oxide	y = 837,932x − 5,475,561 0.996	82 ± 10 ^a^	87 ± 4	7 ^a^	13 ^a^	0.07	0.20	70
		86 ± 6 ^b^		4 ^b^	7 ^b^			
		91 ± 10 ^c^		7 ^c^	11 ^c^			
		88 ± 8 ^d^		3 ^d^	9 ^d^			
Lycopsamine	y = 210,922x + 609,225 0.997	94 ± 4 ^a^	93 ± 5	9 ^a^	6 ^a^	0.07	0.20	88
		90 ± 10 ^b^		8 ^b^	12 ^b^			
		88 ± 5 ^c^		7 ^c d^	5 ^c^			
		100 ± 4 ^d^		5	4 ^d^			
Lycopsamine *N*-oxide	y = 357,975x + 828,088 0.997	89 ± 10 ^a^	89 ± 3	7 ^a^	11 ^a^	0.07	0.20	86
		89 ± 10 ^b^		5 ^b^	11 ^b^			
		85 ± 9 ^c^		9 ^c^	10 ^c^			
		91 ± 5 ^d^		6 ^d^	6 ^d^			
Retrorsine	y = 230,507x + 343,896 0.996	98 ± 5 ^a^	95 ± 3	7 ^a^	5 ^a^	0.07	0.20	96
		94 ± 8 ^b^		4 ^b^	9 ^b^			
		96 ± 8 ^c^		6 ^c^	8 ^c^			
		92 ± 6 ^d^		6 ^d^	7 ^d^			
Retrorsine *N*-oxide	y = 77,663x + 430,608 0.997	99 ± 7 ^a^	94 ± 4	10 ^a^	7 ^a^	0.07	0.20	84
		90 ± 7 ^b^		6 ^b^	7 ^b^			
		94 ± 8 ^c^		4 ^c^	9 ^c^			
		91 ± 4 ^d^		4 ^d^	5 ^d^			
Scopolamine	y = 276,494x + 871,774 0.999	96 ± 8 ^a^	97 ± 1	8 ^a^	7 ^a^	0.03	0.10	91
		97 ± 7 ^b^		5 ^b^	6 ^b^			
		98 ± 6 ^c^		9 ^c^	9 ^c^			
		97 ± 5 ^d^		3 ^d^	5 ^d^			
Senecionine	y = 435,823x + 2,390,060 0.995	88 ± 7 ^a^	93 ± 3	9 ^a^	8 ^a^	0.07	0.20	80
		94 ± 7 ^b^		11 ^b^	7 ^b^			
		96 ± 8 ^c^		8 ^c^	8 ^c^			
		93 ± 6 ^d^		7 ^d^	6 ^d^			
Senecionine *N*-oxide	y = 97,658x + 45,806 0.998	83 ± 8 ^a^	89 ± 4	6 ^a^	9 ^a^	0.07	0.20	80
		91 ± 7 ^b^		4 ^b^	7 ^b^			
		91 ± 6 ^c^		8 ^c^	7 ^c^			
		91 ± 4 ^d^		6 ^d^	5 ^d^			
Seneciphylline	y = 274,323x + 1,192,436 0.995	95 ± 5 ^a^	92 ± 6	6 ^a^	6 ^a^	0.07	0.20	88
		98 ± 6 ^b^		8 ^b^	6 ^b^			
		91 ± 3 ^c^		4 ^c^	3 ^c^			
		85 ± 6 ^d^		6 ^d^	7 ^d^			
Seneciphylline *N*-oxide	y = 79,121x + 522,879 0.993	92 ± 8 ^a^	94 ± 3	9 ^a^	9 ^a^	0.07	0.20	87
		91 ± 7 ^b^		3 ^b^	8 ^b^			
		95 ± 5 ^c^		7 ^c^	5 ^c^			
		97 ± 4 ^d^		4 ^d^	4 ^d^			
Senecivernine	y = 453,180x + 1,717,233 0.998	87 ± 6 ^a^	91 ± 3	10 ^a^	7 ^a^	0.07	0.20	85
		90 ± 10 ^b^		10 ^b^	11 ^b^			
		94 ± 7 ^c^		7 ^c^	8 ^c^			
		92 ± 4 ^d^		7 ^d^	5 ^d^			
Senecivernine *N*-oxide	y = 97,658x + 45,806 0.998	84 ± 6 ^a^	89 ± 3	6 ^a^	7 ^a^	0.07	0.20	80
		92 ± 7 ^b^		5 ^b^	7 ^b^			
		90 ± 4 ^c^		5 ^c^	4 ^c^			
		90 ± 5 ^d^		6 ^d^	6 ^d^			
Senkirkine	y = 194,612x − 512,493 0.999	99 ± 10 ^a^	94 ± 5	6 ^a^	10 ^a^	0.07	0.20	80
		96 ± 8 ^b^		8 ^b^	8 ^b^			
		88 ± 9 ^c^		4 ^c^	10 ^c^			
		93 ± 10 ^d^		4 ^d^	11 ^d^			

Intra-day precision: six extracts of an infusion sample spiked with the analytes at a known concentration level (*n* = 6) analyzed on the same day; Inter-day precision: three extracts of an infusion sample spiked with the analytes at a known concentration level analyzed throughout three different days (*n* = 9); Recovery: nine samples spiked with the analytes at a known concentration level (*n* = 9) subjected to the extraction procedure; ME: matrix effect; MDL: method detection limit; MQL: method quantification limit. ^a^ First spiked level (0.1 µg/L of flower infusion for atropine and scopolamine and 0.2 µg/L of flower infusion for PAs); ^b^ Second spiked level (0.2 µg/L of flower infusion for atropine and scopolamine and 2 µg/L of flower infusion for PAs); ^c^ Third spiked level (2 µg/L of flower infusion for atropine and scopolamine and 4 µg/L of flower infusion for PAs); ^d^ Fourth spiked level (10 µg/L of flower infusion for atropine and scopolamine and 20 µg/L of flower infusion for PAs). * Expressed in ng of PAs/TAs per mL of edible flower infusion.

## Data Availability

The original contributions presented in the study are included in the article/Supplementary Materials, further inquiries can be directed to the corresponding author.

## Supplementary Materials

The following supporting information can be downloaded at: https://www.mdpi.com/article/10.3390/foods13111740/s1, Table S1: Food alerts reported between 2021 and 2023 in edible flowers (data collected from the Rapid Alert System for Food and Feed (RASFF) window [12]; Table S2: Retention time and mass spectrum parameters of the TAs and PAs. Figure S1: Recovery percentages (*n* = 3) for TAs and PAs with PS/DVB cartridges after carrying out the µSPEed^®^ extraction using a jasmine flower infusion spiked with 0.2 ng/mL of a standard solution containing all the target analytes (a) loading 500 µL of sample, 5, 3 and 2 cycles (b) loading 100 µL of sample, 7, 5 and 3 cycles.
