# Supplementary material for: Simultaneous Determination of 23 Pyrrolizidine and Tropane Alkaloids in Infusions from Dry Edible Flowers Using Optimized μSPEed® Microextraction Prior to Their Analysis by UHPLC-IT-MS/MS
Source: Foods. 2024 Jun 1;13(11):1740. doi: 10.3390/foods13111740 (PMC11171954; doi:10.3390/foods13111740)
Supplement: Supplementary file 1 [file foods-13-01740-s001.zip › foods-3010974-supplementary.pdf]

Supplementary Material

Simultaneous determination of 23 pyrrolizidine and tropane alkaloids in infusions from dry edible flowers using optimized µSPEed® microextraction prior to their analysis by UHPLC-MS/MS

Begoña Fernández-Pintor[1], Sonia Morante-Zarcero[1]*, Isabel Sierra[1,2]*

[1] Departamento de Tecnología Química y Ambiental, E.S.C.E.T, Universidad Rey Juan Carlos, C/ Tulipán s/n, 28933 Móstoles, Madrid, Spain

[2] Instituto de Investigación de Tecnologías para la Sostenibilidad. Universidad Rey Juan Carlos, C/ Tulipán s/n, 28933 Móstoles, Madrid, Spain

* Corresponding authors: I. Sierra, S. Morante-Zarcero

E-mail addresses: isabel.sierra@urjc.es; sonia.morante@urjc.es

Table S1. Food alerts reported between 2021 and 2023 in edible flowers (data collected from the Rapid Alert System for Food and Feed (RASFF) window) [12].

Subject	Notification type and date	Reference	Notification basis	Notified by	Countries concerned	Risk and action taken	Distribution status
Tetrahydrocannabinol (THC) in hemp flower tea from Germany, via Austria (FOOD)	Alert 05/05/2023	2023.2985	Official control on the market	Germany	Austria, Germany	Serious Withdrawal from the market	No distribution from notifying country
Unauthorized substance chlorpyrifos ethil in hibiscus flower from Nigeria	Information for attention 23/03/2023	2023.1983	Border control – consignment detained	Italy	Italy, Nigeria, Serbia	Potentially serious Informing authorities and withdrawal from recipient(s)	Distribution restricted to notifying country
Unauthorized substance chlorpyrifos in organic daisy flower from Albania	Information notification follow-up 24/03/2023	2023.1697	Company's own check	France	Albania, France, Germany, Spain	Potentially serious Withdrawal from the market and recall from costumers	Distribution to other member countries
Unauthorized substance chlorpyrifos and thiophanate methyl in chamomile flower	Information for attention 10/03/2023	2023.1644	Company's own check	France	Egypt, France, Germany, INFOSAN	Potentially serious Recall from consumer and withdrawal from recipient(s)	Product (presumably) no longer on the market
Unauthorized novel food *Clitoria* flowers *ternatea* in alcoholic drink (gin)	Information notification follow-up 06/02/2023	2023.0862	Official control on the market	Spain	Belgium, Germany, Italy, Netherlands, South Africa, Spain	Potential risk None	Withdrawal from the market
E-trade of bread prepared with an herbal tea of dried flowers of *Clitoria ternatea* (unauthorized novel food)	Information notification follow-up 06/01/2023	2023.0125	Official control on the market	Spain	Italy, Spain	Undecided None	Product traded online

Gin with *Clitoria ternatea* flowers (unauthorized novel food) from Portugal	Information notification for follow-up 05/01/2023	2023.0116	Official control on the market	Spain	Austria, Belgium, Denmark, Germany, Italy, Luxembourg, Netherlands, Portugal, Spain	Potential risk None	Information on distribution not (yet) available
Salmonella Typhimurium in organic linden flower infusion from Bulgaria, via Austria	Information notification for follow-up 17/02/2021	2021.0827	Official control on the market	Finland	Austria, Bulgaria, Finland, Germany, Italy, Poland, Switzerland	Withdrawal from the market	No distribution from notifying country

Table S2. Retention time and mass spectrum parameters for the TAs and PAs determination by UHPLC-IT-MS/MS.

Analite	Retention time[1] (min)	Precursor ion (m/z)	Isolation (width)	Reaction cut-off	Fragmentation amplitude	Product ions (m/z)
Intermedine	5.3	300	4.0	90	0.7	137.89* – 119.96 – 94.03
Lycopsamine	5.6	300	4.0	90	0.7	137.89* – 94.03 – 119.96
Europine	5.9	330	4.0	120	0.8	253.99* – 137.93 – 155.94
Europine N-oxide	6.6	346	4.0	120	0.8	328.13* – 171.00 – 270.00 – 255.00
Scopolamine	6.7	304	4.0	90	1.0	155.95* – 137.94
Intermedine N-oxide	6.9	316	4.0	100	0.8	171.92* – 137.98 – 225.90
Lycopsamine N-oxide	7.2	316	4.0	100	0.8	171.92* – 137.98 – 225.90
Retrorsine	8.1	352	4.0	120	0.8	324.16* – 276.05 – 333.13
Retrorsine N-oxide	8.2	368	4.0	130	0.9	246.03* – 340.25
Seneciphylline	8.4	334	4.0	110	0.8	306.07* – 262.93 – 228.00
Heliotrine	8.5	314	4.0	100	0.7	137.94* – 119.89 – 155.97
Seneciphylline N-oxide	9.1	350	4.0	100	0.8	322.11* – 288.05
Heliotrine N-oxide	9.3	330	4.0	120	1.0	172.87* – 227.96 – 298.99
Atropine	9.5	290	4.0	85	0.9	123.99* – 91.04 – 93.04 – 243.18
Senecivernine	9.6	336	4.0	110	0.8	308.10* – 119.94 – 137.96
Senecionine	9.8	336	4.0	110	0.8	308.11* – 119.94 – 137.96
Senecivernine N-oxide	10.0	351.4	4.0	120	0.8	324.14* – 219.97
Senecionine N-oxide	10.5	351.4	4.0	100	1.0	324.14* – 219.97
Echimidine N-oxide	11.5	414	4.0	100	0.6	253.96* – 396.17 – 352.14
Echimidine	11.7	398	4.0	110	0.7	119.95* – 219.97 – 336.17
Senkirkine	12.0	366	4.0	130	0.8	167.91* – 331.17 – 149.92
Lasiocarpine	12.8	412	4.0	100	0.7	336.15* – 219.96 – 119.99 – 305.80
Lasiocarpine N-oxide	13.2	428	4.0	100	0.8	410.21* – 352.10 – 322.26 – 254.01

[1]Chromatography conditions: with the optimized gradient elution: 5 % A (0-0.5 min), 5-10% A (0.5-3.5 min), 10-25 % A (3.5-7.5 min), 25-30 % A (7.5-9.5 min), 30-70 % A (9.5-12.5 min), 70-5 % A (12.5-14.5 min). Methanol containing 0.2 % ammonia as mobile phase A and water containing 0.2 % formic acid as mobile phase B. The flow rate was 0.3 mL/min

*Ions used for quantification

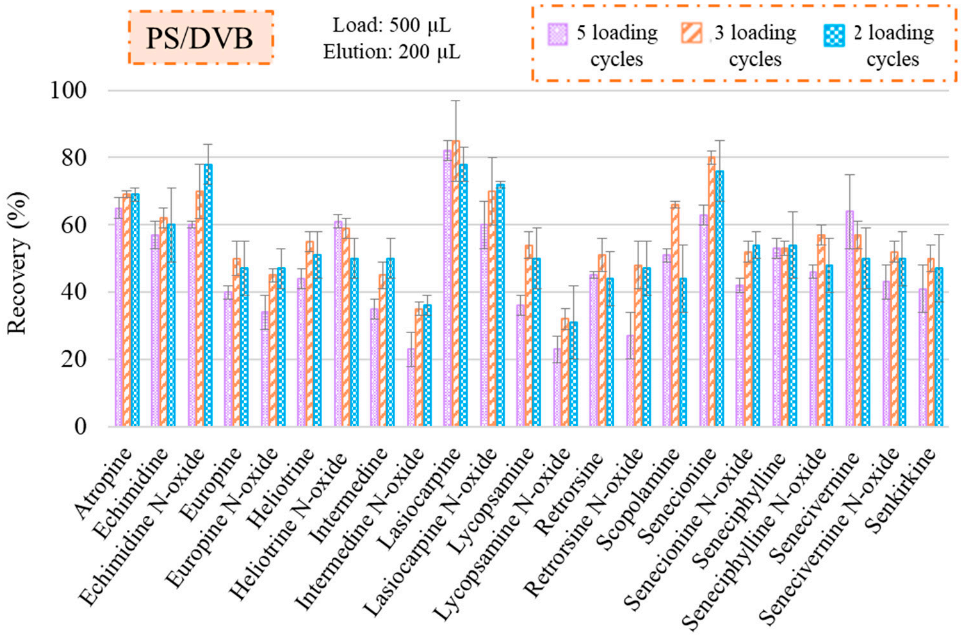

(**a**)

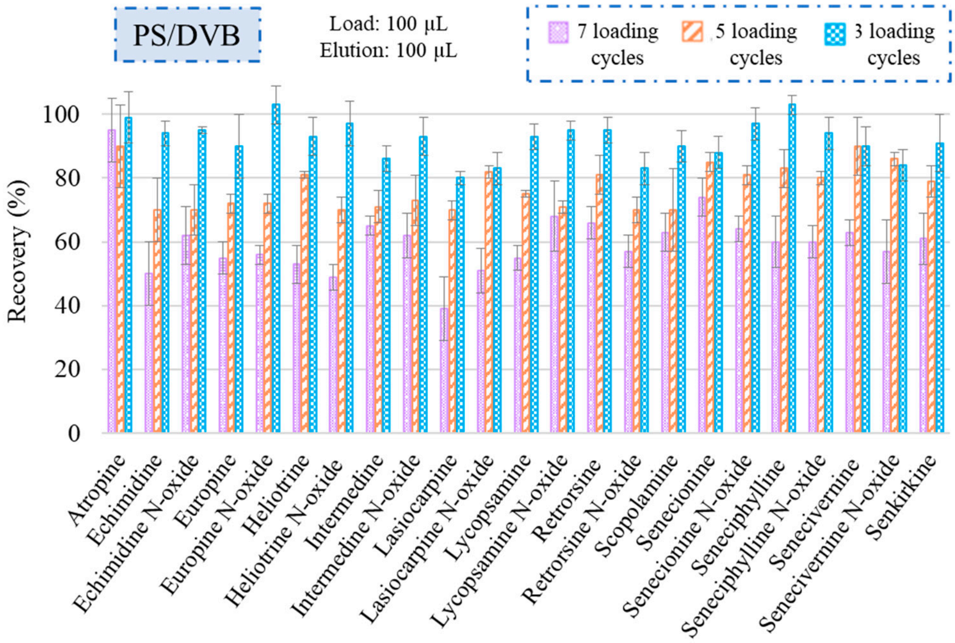

(**b**)

Figure S1. Recovery percentages (n=3) for TAs and PAs with PS/DVB cartridges after carrying out the µSPEed® extraction using a jasmine flower infusion spiked with 0.2 ng/mL of a standard solution containing all the target analytes (a) loading 500 µL of sample, 5, 3 and 2 cycles (b) loading 100 µL of sample, 7, 5 and 3 cycles.
